# Enhancing hospital competitiveness: use of the electronic dashboard for managing the power of executing information systems

**DOI:** 10.1186/1472-6963-14-S2-P15

**Published:** 2014-07-07

**Authors:** Chia-Jung Chen, June-Dong Lin, Chung-Feng Liu

**Affiliations:** 1 Department of Information Systems, Chi Mei Medical Center, Tainan, Taiwan; 2 Department of Information Management, Chia Nan University of Pharmacy and Science, Tainan, Taiwan

## Background

With the increasing demands of a variety of IT support for health care, managing the execution of the information systems (IS) department is increasingly critical but complex. Therefore, the Chi Mei Medical Center has proposed a one-year-project to develop an electronic dashboard (e-Dashboard) for the execution of IS with visual interfaces to present the status of IS services requested by users.

## Materials and methods

A prototype method was adopted. Prior to the programming phase, a series of performance indicators were defined according to an expert panel. Based on “The 4 Disciplines of Execution” [1] as well as an internal speech “To Enhance Competitiveness” [2] by the superintendent of the medical center, Dr Chio, at the 2013 Executive Consensus Seminar, this e-Dashboard focuses on four core principles.

1. Focus on the wildly important

2. Act on the lead measures

3. Keep a compelling scoreboard

4. Create a cadence of accountability

## Results and conclusions

The benefits after introducing the e-Dashboard can be illustrated through three aspects.

### 1. Establish IS performance metrics

In the past, there has been no effective way to measure IS staff’s performance. In this project, we conducted a series of quantitative indicators and presented them in a timely and transparent manner on large wall mounted monitors. This provided direct information for communication between users and IS staff.

### 2. Enhance IS executive power and competitiveness

E-Dashboards can monitor IS performance on daily, weekly, or even monthly timelines and provide drill-down features to browse and analyze the performance status by layers of departments, teams and individuals. As a result, we can effectively control the service performance and then enhance IS execution.

### 3. Help managers make decisions

E-Dashboards can be used to monitor real-time events during operational processes, and to help IS managers obtain instant messages and make effective decisions.

E-Dashboards can help IS managers keep track of instant messages, thereby making the most immediate and correct decisions, and thus enhancing the power of IS execution. Currently, Chi Mei Medical Center is beginning to develop other types of e-dashboards for other purposes such as for monitoring the emergency department’s waiting list. This will help to maximize the effectiveness of the e-Dashboard - not only for the IS department but for the hospital as a whole.
